# Navigating thyroid cancer complexity: the emerging role of EV-derived non-coding RNAs

**DOI:** 10.1038/s41420-025-02411-1

**Published:** 2025-04-04

**Authors:** Meng Jia, Jiawen Liang, Lu Gao, Na Wei, Ye Qin, Qianqian Li, Xintao Wang, Jian Zheng, Hao Wang, Jie Wang, Shuo Wang, Xiubo Lu

**Affiliations:** 1 Department of Thyroid surgery, The First Affiliated Hospital of Zhengzhou University, Zhengzhou, Henan 450052 China; 2 Department of Cardiology, The First Affiliated Hospital of Zhengzhou University, Zhengzhou, Henan 450052 China; 3 Department of Pathology, The First Affiliated Hospital of Zhengzhou University, Zhengzhou, Henan 450052 China

**Keywords:** Cancer microenvironment, Cancer microenvironment

## Abstract

Thyroid cancer (TC), which arises from the epithelial cells of the thyroid gland, is experiencing a significant increase in incidence globally. TC encompasses various subtypes, including papillary, follicular, medullary, and anaplastic thyroid cancers, each with distinct pathological and clinical features. Extracellular vesicles (EVs), are naturally occurring and nanosized lipid bilayers, and can be secreted by almost all cell types. EVs, comprising microvesicles and exosomes, are pivotal in mediating intercellular communication within the tumor microenvironment. Notably, EVs possess unique properties such as stability in circulation and the ability to traverse biological barriers, enhancing their role as carriers of molecular information. EVs carry non-coding RNAs (ncRNAs), including miRNAs, lncRNAs, and circRNAs, which are crucial regulators of gene expression. Recent studies have highlighted the significant role of EV-derived ncRNAs in influencing thyroid cancer progression, metastasis, and immune modulation by mediating intercellular communication within the tumor microenvironment. The expression of EV-derived ncRNAs varies across different stages of thyroid cancer, reflecting potential as biomarkers for diagnosis and targets for therapy. This review delves into the multifaceted roles of EV-ncRNAs in thyroid cancer, emphasizing their impact on tumor growth, metastatic potential, and immune interactions, while also exploring their promising applications in early diagnosis and targeted treatment strategies. Understanding these dynamics is essential for developing innovative interventions to improve patient outcomes in thyroid cancer.

## Fact

1. EV-derived ncRNAs are crucial orchestrators in reshaping thyroid cancer progression.
2. EV-derived ncRNAs can modulate the immune cell composition in the thyroid cancer microenvironment, potentially affecting tumor progression and patient prognosis.
3. The presence of specific EV-derived ncRNAs in blood samples could serve as early indicators of thyroid cancer, even before clinical symptoms appear, improving early diagnosis rates.
4. Inhibiting specific EV-derived ncRNAs that are known to promote drug resistance could enhance the effectiveness of existing treatments, offering a new avenue for targeted therapy in resistant thyroid cancer.

## Open Questions

1. What are the specific molecular pathways through which EV-derived ncRNAs influence tumor cell and stromal behaviors in the thyroid cancer microenvironment?
2. How do levels of specific EV-derived ncRNAs change over the course of thyroid cancer treatment?
3. How can EV-derived ncRNAs be integrated into combination therapies to enhance the efficacy of existing thyroid cancer treatments?

## Introduction

Thyroid cancer (TC) is a malignant tumor derived from the epithelial cells of the thyroid gland and is the fastest increasing solid malignancy in these years [1]. Currently, the main pathological types of TC include papillary thyroid cancer (PTC), follicular thyroid cancer (FTC), medullary TC, and anaplastic thyroid carcinoma (ATC) [2, 3]. Among them, TC with increased incidence is mainly PTC [4]. About 1-2% of TC is ATC, which is the most lethal and treatment-resistant malignancy of the endocrine system [5]. Although the incidence of TC is high with a relatively high survival rate, patients with advanced and severe stages still have no curative measures and need lifelong medication to sustain their lives [6]. Therefore, it is extremely imperative to seek precise molecular markers for the early diagnosis and treatment of TC.

**Fig. 1:**
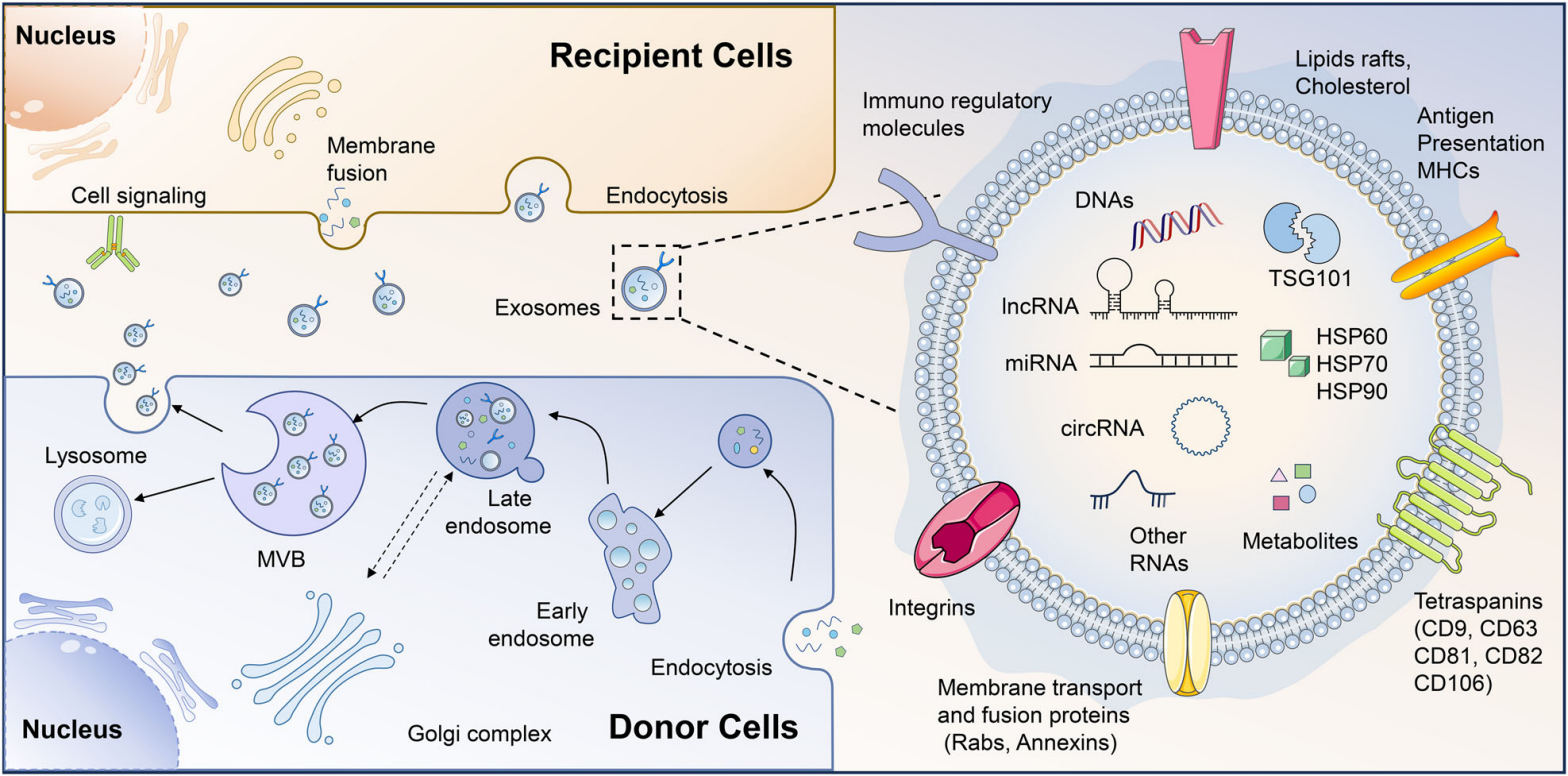
Overview of the biogenesis, secretion, uptake and composition of exosomes. Exosomes are small extracellular vesicles formed through the endocytic pathway, involving stages such as early endosomes, multivesicular bodies (MVBs), and intraluminal vesicles (ILVs), which eventually fuse with the cell membrane to release exosomes into the extracellular space. These vesicles carry a variety of biologically active substances, including DNA, mRNAs, non-coding RNAs, proteins, and lipids. Specific protein markers, such as tetraspanins (CD9, CD63, CD81), are used to identify exosomes. The recipient cells uptake, via different pathways, such as cell signaling, membrane fusion, or endocytosis (left). The featured structure and common components of exosomes (right). lncRNA Long-noncoding RNA, miRNA MicroRNA, circRNA circular RNA, MHC Major histocompatibility complex class.

Under both physiological and pathological conditions, almost all cells are capable of forming and releasing extracellular vesicles (EVs), mainly consisting of microvesicles with diameters of 50 nm-1 μm and exosomes with diameters of 40 nm-160 nm [7]. Exosomes are formed by the endocytic pathway through several steps, including early endosomes, intracellular multivesicular bodies (MVBs), intraluminal vesicles (ILVs), and finally ILVs fuse with the cell membrane and release EVs into the extracellular space [8]. EVs can incorporate a multitude of biologically active substances, including DNA fragments, mRNAs, non-coding RNAs (ncRNAs), proteins, lipids, and small metabolites [9, 10]. EVs produce specific protein biomarkers during formation that are available for the identification of EVs, including tetraspanins (CD9, CD63, CD81), adhesion molecules (integrins, EPCAM), antigen presentation molecules (MHC-I, MHC-II), membrane trafficking and fusion proteins (Rabs, Annexins) **(**Fig. 1) [11, 12].

The tumor microenvironment (TME) is a heterogeneous entity composed of soluble factors, tumor cells, and multiple other groups of cells, including immune cells, stromal cells, angiogenesis-associated cells, and extracellular matrix [13]. Within the TME, extracellular vesicles (EVs) are vehicle ingredients for information interactions, playing a crucial role in regulating thyroid carcinogenesis, TME remodeling, and tumor malignant behaviors [14, 15]. For example, Delcorte et al. analyzed EVs from a mouse model of PTC expressing BRAFV600E, revealing an increased release of EVs with epithelial and immune markers compared to normal thyroid tissue [16]. These EVs, primarily originating from thyrocytes, exhibited deregulated microRNA content as cancer progressed. PTC-derived EV-microRNAs might contribute to creating a permissive immune microenvironment, potentially influencing tumor progression and recurrence. In PTC, some tumors exhibit aggressive behavior through epithelial-mesenchymal transition (EMT), where epithelial cells transform, reducing adhesion and increasing motility [17]. The tumor microenvironment, enriched with factors like hypoxia and HMGB1, significantly influences this transformation. Hypoxia alters cell phenotypes to enhance metastasis, while EVs mediate intercellular communication by transferring molecules that modify recipient cell protein expression.

Non-coding RNAs (ncRNAs) are important components of the human genome and traditionally belong to endogenous RNA molecules that do not encode proteins [18, 19]. NcRNAs are comprised of multiple types and perform different functions, such as microRNAs (miRNAs), long non-coding RNAs (lncRNAs), circular RNAs (circRNAs), small nuclear RNAs (snRNAs), and PIWI-interacting RNAs (piRNAs) [20, 21]. NcRNAs are no longer perceived as bystanders, but as key regulatory molecules that mediate cellular activity through specific regulation of gene expression, including chromatin remodeling, transcriptional regulation, and post-transcriptional modifications [22]. Multiple types of ncRNAs are involved in tumor initiation, oncogenesis, epithelial-mesenchymal transition (EMT) processes, and drug resistance. Various internal and external factors stimulate and lead to spatiotemporal changes in tissue-specific EV-derived ncRNAs (EV-ncRNAs), which are key drivers of TC progression [23, 24].

**Fig. 2:**
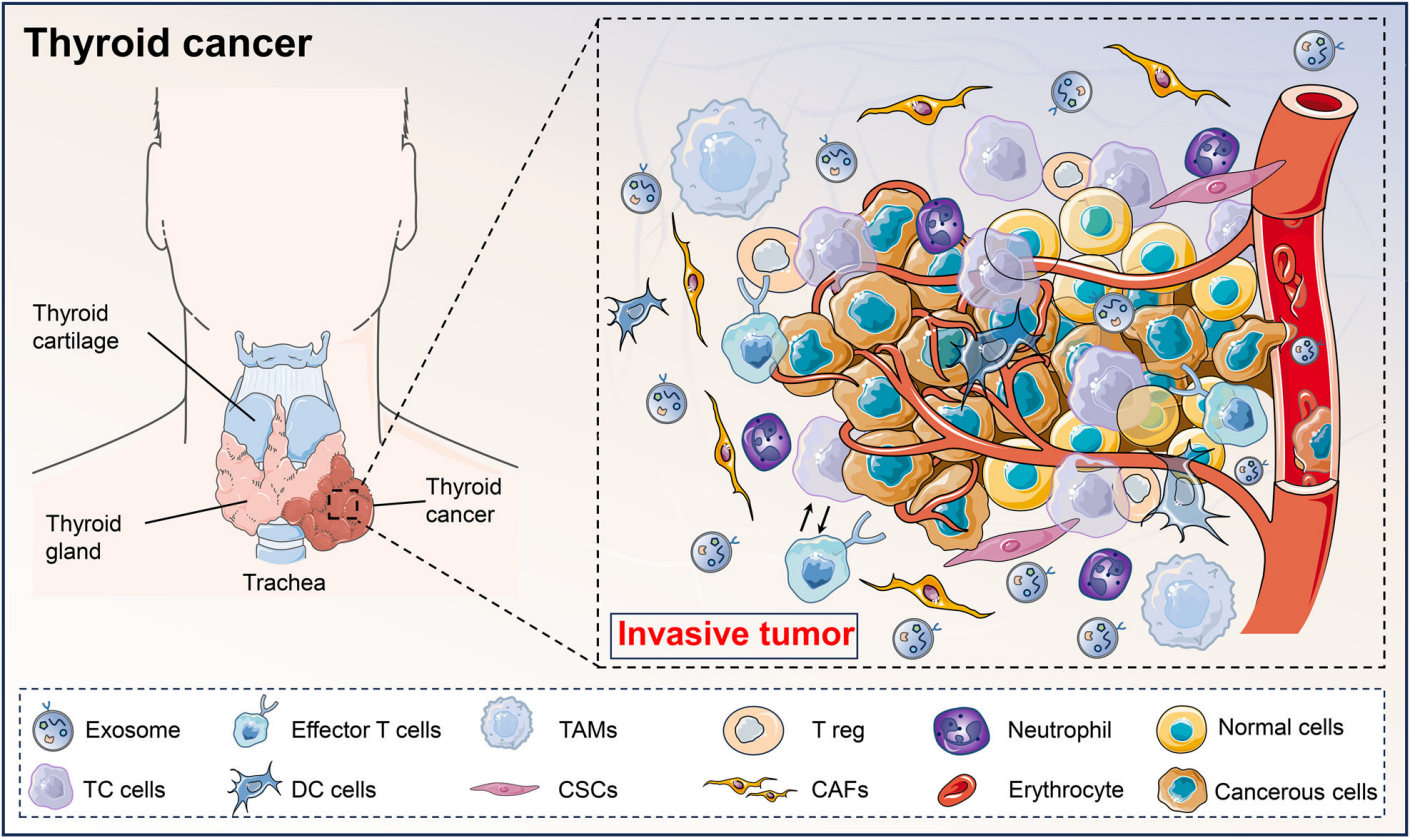
EVs are included in the TC tumor microenvironment. The tumor microenvironment of TC is a heterogeneous and dynamic entity with multiple factors and cellular composition. These cells include tumor cells (TC cells, CSCs), immune cells (DC cells, T cells, TAMs, neutrophils), stromal cells (CAFs), as well as exosomes of multiple cellular origin as carriers of intercellular interactions. A key aspect of this environment is the presence of extracellular vesicles (EVs) originating from multiple cell types, which serve as crucial mediators of intercellular communication. Within these EVs, non-coding RNAs (ncRNAs) act as influential regulators of gene expression. EV-derived ncRNAs can significantly impact TC progression by modulating processes such as tumor proliferation, invasion, migration, epithelial-mesenchymal transition (EMT), and metastasis, by inducing gene silencing through epigenetic modifications. EVs extracellular vesicles, TC thyroid cancer, DC dendritic cell, TAMs tumor-associated macrophages, CSCs cancer stem cells, CAFs cancer-associated fibroblasts, EMT epithelial-mesenchymal transition.

EVs can be secreted by almost all cells in TC TME and can transport a variety of characteristic nucleic acid substances of parental cell origin [25]. In particular, TC cell-derived EVs are able to deliver ncRNAs, represented by miRNAs, lncRNAs, and circRNAs, to recipient cells and thus influence their biological functions. Moreover, the abundance of tumor-derived EVs is very high, which is because TC cells can continuously secrete EVs and enter the circulatory system and are present in various body fluids, resulting in a much higher EV production than normal cells [26]. Based on the above considerations, EV-ncRNAs play an important regulatory role in a variety of pathophysiological processes in TC, and may be vital targets for adjuvant diagnosis, targeted therapy, and prognostic assessment of TC (Fig. 2).

Therefore, based on the above-mentioned research status, this review mainly systematically summarizes the impact of EV-ncRNAs, including miRNAs, lncRNAs, and circRNAs, on the biological behavior and the value of diagnosis and treatment of TC. In-depth studies based on EV-ncRNAs will provide a strong theoretical basis for accurate diagnosis, stratification, and targeted therapy of TC, which will help to improve the survival quality and reduce the disease-related mortality of patients.

## NcRNAs from EVs in TC growth and metastasis

The interaction of TC cells with stromal cells affects the distinctive changes in the proteome and genome of released exosomes, an event that can have some impact on EVs and TC progression. For example, the interaction of TC cells with fibroblasts produced a significantly greater abundance of EVs than their respective cells alone, and these EVs could substantially contribute to MMP2 activation and promote ECM degradation, thus leading to TC progression [27]. After entering the recipient cells, EV-ncRNAs can contribute to the altered behavior of tumor cells by regulating the expression and function of target genes [28].

### EV-derived miRNAs

MiRNAs are a class of short-stranded ncRNAs that can bind to target mRNAs to achieve gene silencing and translation inhibition [29, 30]. The miRNA biogenesis and processing machinery is an influential contributor to the regulation of TC processes [31, 32]. EV-mediated miRNAs, through their interactive transfer in tumor proliferation, angiogenesis, metastasis, and chemoresistance, emerge as cutting-edge diagnostic biomarkers and therapeutic targets across various cancers [33].

MiR-146b and miR-222 were overexpressed in PTC cell exosomes, and these exosomes had a negative proliferative impact on both TPC-1 and NTHY cells [34]. Compared with normal patients, miR-423-5p was significantly elevated in the serum of PTC patients [35]. At the cellular level, the TPC-1 cell exosomal miR-423-5p effectively promoted the migration and invasive functions of PTC cells. Under hypoxic conditions, miR-21-5p expression in PTC serum and BCPAP cell exosomes showed an upregulation trend, a phenomenon that enhanced the angiogenic effect [36]. Particularly, exosomal miR-21-5p directly targeted and repressed TGFBI and COL4A1, leading to increased endothelial tube formation, thus revealing a critical role of the miR-21-5p/TGFBI and miR-21-5p/COL4A1 regulatory axis in PTC angiogenesis in exosomes under hypoxic conditions. Hypoxia-derived exosomal miR-221-3p was upregulated in PTC and played a key role in promoting tumor progression by enhancing cell proliferation, migration, invasion, and EMT through targeting ZFAND5 [37]. Inhibition of miR-221-3p reduced tumor growth in PTC models, highlighting its potential as a biomarker for diagnosis and prognosis, as well as a target for therapeutic intervention. Additionally, Powell et al. confirmed that miR-210-3p levels were increased under low oxygen conditions in the ATC cell line SW1736. And, miR-210-3p was released alongside extracellular vesicles and AGO2, indicating its potential as a cellular and extracellular hypoxia biomarker [38].

### EV-derived lncRNAs

LncRNAs are ncRNAs greater than 200 nucleotides in length that regulate the expression of miRNAs and downstream genes [39, 40]. LncRNAs are involved in transcriptional, translational, and post-translational modifications and are highly correlated with tumor-related malignant phenotypes [41]. CDKN2B-AS1 is an ncRNA that promotes TC and is highly expressed in cancer stem cells (CSCs) and CSC-derived exosomes [42]. CSC-derived exosomal CDKN2B-AS1, promoted TC cell proliferation, migration, invasion, and decreased protein expression of N-cadherin and Vimentin as well as TGF-β1/Smad2/3 signaling [43]. This indicated that CSC-derived exosome CDKN2B-AS1 stabilized CDKN2B and promoted TC progression through TGF-β1/Smad2/3 signaling. DOCK9-AS2 was significantly elevated in PTC and plasma exosomes by bioinformatic analysis [44]. Functionally, PTC-CSC-derived exosomal lncRNA DOCK9-AS2 could enhance CTNNB1 expression and activate the Wnt/β-catenin pathway by binding SP1, as well as targeting miR-1972/CTNNB1. Ultimately, exosomal lncRNA DOCK9-AS2 contributed to the exacerbation of PTC through the classical lncRNA-miRNA binding pattern. Another study also showed that lncRNAs transported by CSC exosomes, particularly linc-ROR, were able to initiate EMT and boosted tumor seed cell propagation and colonization of distant metastatic niches [45].

### EV-derived circRNAs

**Fig. 3:**
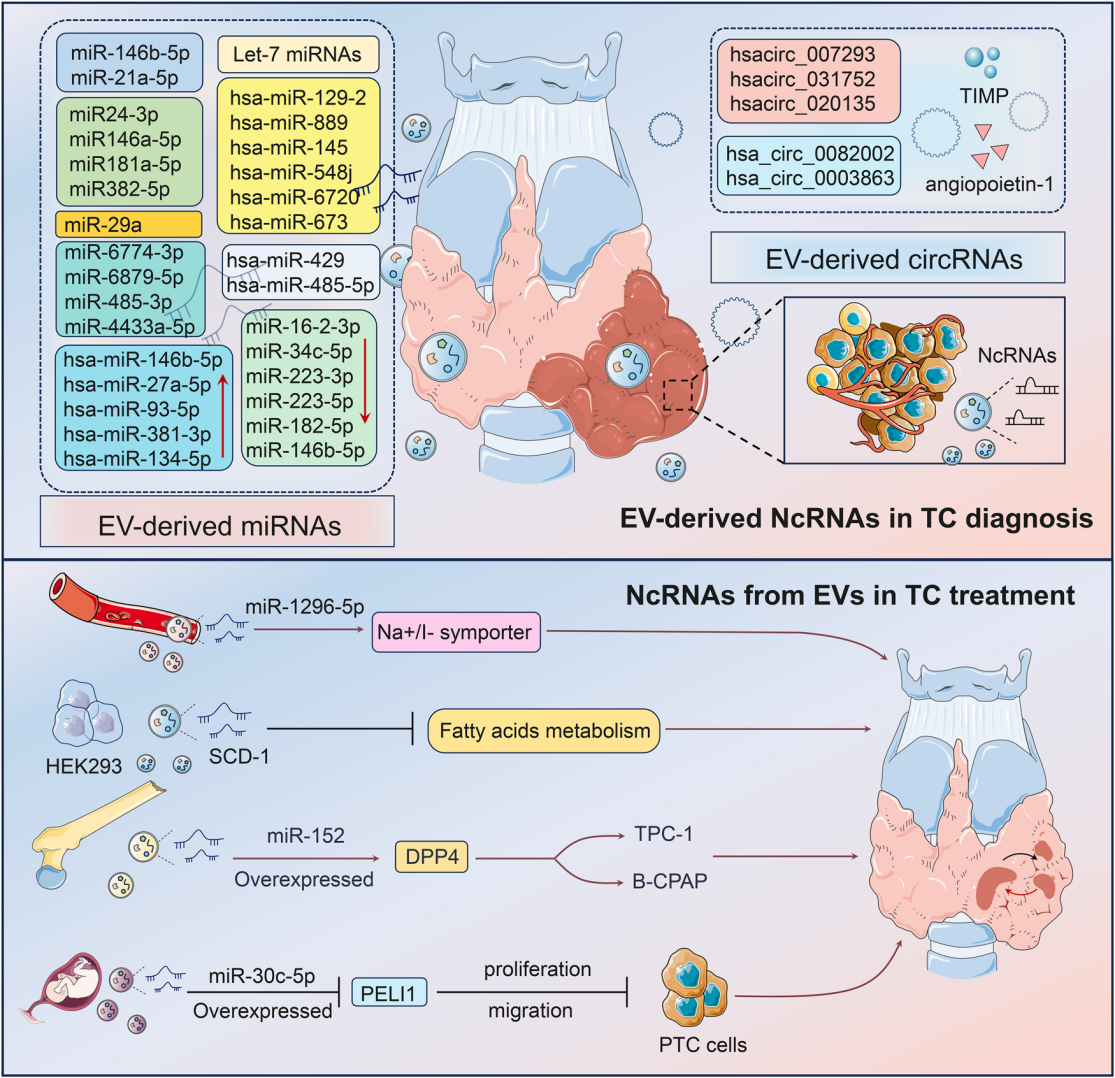
The contributions of tumor-derived EV-ncRNAs to TC progression. EV-ncRNAs play crucial roles in the growth and metastasis of TC by mediating intercellular communication within the tumor microenvironment. EVs carry various ncRNAs, including miRNAs, lncRNAs, and circRNAs, which significantly influence oncogenic processes. For instance, miRNAs such as miR-146b and miR-222 are overexpressed in PTC cell exosomes, negatively affecting cell proliferation. MiR-423-5p, elevated in PTC serum, enhances cell migration and invasion, while miR-21-5p, upregulated under hypoxic conditions, promotes angiogenesis by targeting TGFBI and COL4A1. Additionally, miR-221-3p facilitates proliferation, migration, invasion, and epithelial-mesenchymal transition (EMT) by targeting ZFAND5. LncRNAs like CDKN2B-AS1, found in cancer stem cell-derived exosomes, boost TC cell proliferation and invasion through TGF-β1/Smad2/3 signaling, whereas DOCK9-AS2 activates the Wnt/β-catenin pathway, exacerbating PTC progression. CircRNAs, such as circ007293, enriched in PTC patient serum exosomes, promote EMT, invasion, and proliferation by regulating the miR-653-5p/PAX6 axis. These EV-ncRNAs are integral to modifying the tumor microenvironment and driving TC progression.

CircRNAs are a class of ncRNA molecules covalently bonded to form a ring structure with tissue-restricted and cancer-specific expression patterns [46]. CircRNAs can mediate intercellular communication via EV delivery and function as gene regulators primarily as competing endogenous RNAs (ceRNAs) for miRNAs [47]. Lee et al. verified that circ007293 was enriched in exosomes from the serum of PTC patients and cell culture media [48]. Exosomal circ007293 fostered EMT, invasion, and proliferation effect of PTC cells by targeting and regulating the miR-653-5p/PAX6 axis. Thus, exosomal circ00729 was an important molecule for promoting the PTC process and potential therapeutic biomarker (Fig. 3).

## NcRNAs from EVs in TC immune regulation

EVs perform important roles in either innate or acquired immunity, including antigen presentation, amplification of inflammatory signals, and lymphocyte development and activation [49]. Immune cells can be suppressively domesticated by tumor cells through direct physical contact or secreted factor modulation [50, 51]. EVs of tumor origin harbor the immune escape and immunogenic properties of parental tumor cells and thereby drive malignant biological alterations occurring in the tumor [52].

Macrophages are a highly diverse and heterogeneous category of cell populations that can generally be classified as anti-tumor M1 phenotype or pro-tumor M2 phenotype [53, 54]. Qiao et al. showed that exosomal miR-655-3p was significantly downregulated in the serum of PTC patients [55]. Exosomal miR-655-3p could suppress PTC growth, invasion, and M2 macrophage polarization in the xenograft model, by inhibiting CXCR4 expression. This study posed that exosome-miR-655-3p/CXCR4 was a novel targeting axis in treating PTC. Li et al. revealed that PTC-derived exosomal miR-519e-5p was markedly elevated in the circulatory system of distant metastatic PTCs [56]. Specifically, miR-519e-5p promoted a malignant phenotype by activating the Wnt signaling pathway and contributed to CD8 + T-cell exhaustion by inhibiting the Notch signaling pathway. Thus, the transfer of miR-519e-5p via circulating exosomes strengthened tumor malignancy and CD8 + T-cell exhaustion in metastatic PTC.

## NcRNAs from EVs in TC diagnosis

Most of the TCs are silent and the early symptoms are not noticeable [57]. Early detection and diagnosis can improve the cure rate of TC. In addition to conventional pathological diagnosis, hematological examination, and imaging methods, EVs and EV-derived ncRNAs can be stably and characteristically present in the blood, urine, and saliva of TC patients, therefore becoming novel circulating tumor markers [58]. Standardized methods for isolation, preparation, and precise detection of EV-ncRNAs are essential for differential diagnosis of TC.

### EV-derived miRNAs in TC diagnosis

TC is a relatively common disease, and pathological specimens of the thyroid gland account for a substantial amount of the routine pathology work. Therefore, the diagnosis of thyroid disease and benign and malignant differentiation is of great importance. For instance, Delcorte et al. demonstrated that miR-146b-5p and miR-21a-5p exhibited distinct differences in abundance in purified plasma-derived EVs from patients with PTC and benign diseases [59]. Zabegina et al. presented an interesting study in which thyroid peroxidase (TPO)-positive EVs were first isolated and validated using an immunomagnetic bead method, and then RT-qPCR was used to quantify the characteristic Let-7 family members in these EVs expression [60]. It was revealed that the expression properties of Let-7 miRNAs in TPO(+) EVs could differentiate follicular adenomas and FTC. Using RNA-seq technology, Liang et al. showed that the expression of plasma exosomal miR-16-2-3p, miR-34c-5p, miR-223-3p, miR-223-5p, miR-182-5p, and miR-146b-5p were reduced PTC compared to benign thyroid nodules [61]. The signature consisting of miR-16-2-3p, miR-223-5p, miR-101-3p, and miR-34c-5p was qualified to discriminate benign and malignant thyroid nodules. Wang et al. identified that hsa-miR-146b-5p, hsa-miR-27a-5p, hsa-miR-93-5p, and hsa-miR-381-3p and hsa-miR-134-5p were upregulated in the serum exosomes of PC patients [62]. It is proposed that exosomal hsa-miR-27a-5p could effectively distinguish parathyroid carcinoma (PC) from parathyroid adenoma (PA), and therefore can be used for preoperative identification of PC and PA subjects.

Serum exosomal miR-29a could discriminate between PTC and normal controls, and its reduced expression was strongly associated with a higher risk of recurrence, worse prognosis, and TNM staging [63]. This suggested that serum exosomal miR-29a possessed high predictive efficacy in the diagnosis and prognosis of PTC. Capriglione et al. showed that serum miR24-3p, miR146a-5p, miR181a-5p, and miR382-5p were differentially expressed in exosomes from PTC patients compared to healthy individuals [64]. Therefore specific miRNAs were selectively encapsulated in exosomes, influencing immune responses and potentially serving as biomarkers for PTC, although their role in predicting lymph node metastasis remains inconclusive.

The abundance of EV-ncRNA in different body fluids of TC patients, especially blood, is characterized by differential expression in different disease stages. Therefore single EV-ncRNAs may be considered potential diagnostic, prognostic, or therapeutic evaluation markers. In addition, combined or predictive models based on multiple EV-ncRNAs enable precise prognosis determination in addition to the evaluation of TME features, immune infiltration, and genetic changes. The study by Dai et al. highlighted that plasma exosomal miR-485-3p and miR-4433a-5p might hold excellent potential for the diagnosis of PTC [65]. Moreover, plasma exosome miR-485-3p enabled the distinguishing of high-risk and low-risk PTC. Xin et al. constructed a risk scoring system based on 6 miRNAs, hsa-miR-129-2, hsa-miR-889, hsa- miR-145, hsa-miR-548j, hsa-miR-6720, and hsa-miR-673, and confirmed that this model was a predictor of prognosis in patients with PTC [66]. Chen et al. used miRNA microarrays to analyze and model plasma exosomal miRNAs [67]. They successfully identified plasma exosomal miR-6774-3p and miR-6879-5p and their combinations, possessing high AUC prediction values and higher performance than total miRNAs isolated from plasma. Thus, plasma exosomal miR-6774-3p and miR-6879-5p and their combinations could be used as potential diagnostic markers of lymph node metastasis (LNM) in PTC. However, it is noteworthy that Maggisano et al. identified the secretory status of miR24-3p, miR146a-5p, miR181a-5p, and miR382-5p in the exosomes of PTC patients [68]. They highlighted the need for data support and careful handling of circulating exosomal miRNAs in predicting lymph node metastasis.

Of particular interest, Wise et al. brought TC cells to the international space station and analyzed the impact of microgravity after a few days on the profile of TC cell-secreting miRNAs by microarray [69]. The results identified more than 100 differentially expressed miRNAs, such as hsa-miR-429 and hsa-miR-485-5p, suggesting that space gravity or other stressful environmental stress alterations are also important factors in promoting altered genetic traits in TC tumors. Besides, weightlessness may have reversible or non-reversible effects not only on TC cells but also on normal cells or changes at the physiological level.

### EV-derived circRNAs in TC diagnosis

In addition to miRNAs, circRNAs have emerged as promising biomarkers in TC diagnosis. CircRNAs have unique stability due to their covalently closed loop structure, making them resistant to exonucleases [46]. Besides, circRNAs often exhibit tissue-specific expression patterns, which can enhance the specificity of cancer diagnostics [70]. Yang et al. displayed the presence of specific differentially expressed circRNAs in PTC and benign thyroid goiter serum exosomes, including hsacirc_007293, hsacirc_031752, and hsacirc_020135 [71]. These circRNAs are involved in the regulation of TC-related pathways and may serve as biomarkers for differential diagnosis. Dai et al. investigated the diagnostic potential of exosomal circRNAs, specifically hsa_circ_0082002 and hsa_circ_0003863, in papillary thyroid carcinoma (PTC). Interestingly, these circRNAs were significantly elevated in PTC patients with correlations to lymph node metastasis and vascular invasion [72].

There are other proteins, nucleic acids, and small molecule components of body fluid exosomes that may possess functions for early screening, disease detection, and prognostic evaluation [73]. For example, Wang et al. suggested that urinary exosomal TIMP and angiopoietin-1 represented promising preoperative biomarkers for well-differentiated TC. Most of the above studies are retrospective, the practical application in the clinical setting needs to be determined by further studies in large cohorts and long-term follow-up. Moreover, integrating EV-ncRNA profiles with existing diagnostic frameworks could improve the accuracy of distinguishing between benign and malignant thyroid conditions, as well as predicting patient outcomes. The development of comprehensive multi-marker panels and predictive models based on EV-ncRNAs could revolutionize personalized medicine approaches in TC management.

## NcRNAs from EVs in TC treatment

When tumors undergo interventions such as chemotherapy, radiotherapy, and immunotherapy, many tumor cells, immune cells, and other stromal cells will produce corresponding alterations in exosomes, as manifested by secreted content, and genomic and proteomic remodeling [74–76]. These alterations in EVs can lead to responsive changes that enhance or diminish treatment. Although differentiated PTC generally presents a better prognosis and lower mortality, a proportion of cases exhibit progressive behavior and become radioiodine refractory (RAIR) PTC, and the survival of this group of patients is significantly shorter [77]. Li et al. identified that exosomal miR-1296-5p was upregulated in RAIR cell lines, the plasma, and the tissues in RAIR PTC patients [78]. They hypothesized that circulating exosomal miR-1296-5p might be engaged in the mechanism of RAIR PTC pathogenesis by directly targeting Na + /I− symporter (NIS).

As nanoscale vesicles, exosomes have long been shown to be excellent vehicles due to their low immunogenicity, circulating properties, high biosafety, and ability to upload active molecules [79, 80]. For example, Wang et al. reported that there was a higher SCD-1 expression pattern in ATC cell lines compared to normal thyroid cell line [81]. Then, they constructed a therapeutic platform by encapsulating SCD-1 siRNA in exosomes obtained from HEK293 cells. This platform was a feasible strategy that enhanced the death of ATC cells by regulating fatty acid metabolism and reactive oxygen species (ROS) level. Wang et al. engineered the HEK-293T exosome-based targeted delivery platform (Dox@iRGD-Exos-131I) with radionuclides and chemotherapy ability [82]. This study demonstrated a strong capability for targeting tumors and significantly inhibiting tumor growth. This provides a highly efficient and multifunctional vesicle-level nano-tool for ATC treatment.

**Fig. 4:**
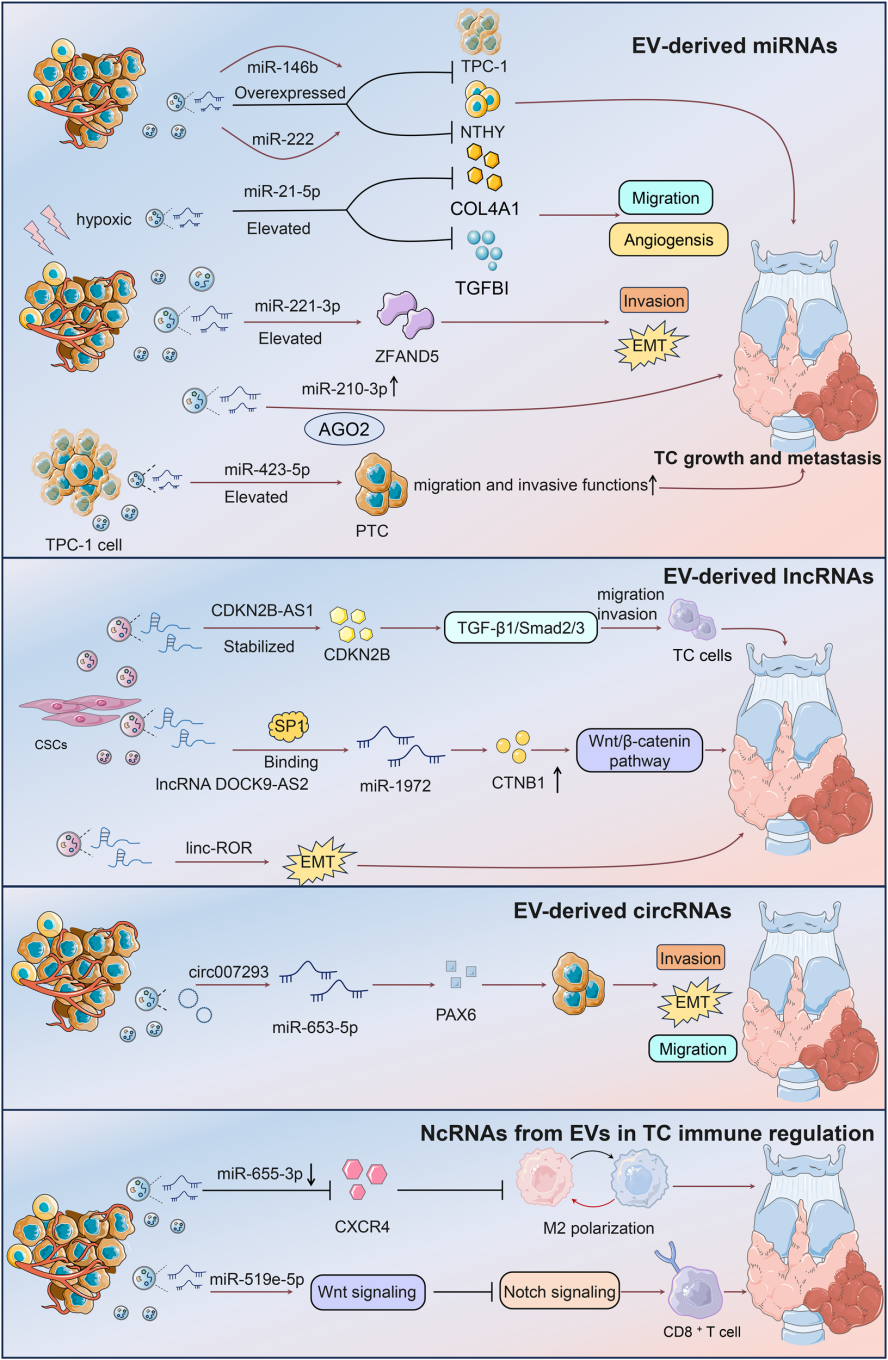
The potential of EV-ncRNAs as biomarkers and therapeutic agents in TC. In diagnosis, EV-derived miRNAs, such as miR-146b-5p, miR-21a-5p, and Let-7 family members, show differential expression in patients with PTC versus benign conditions, offering promising biomarkers for early detection and differentiation. Additionally, circRNAs like hsa_circ_0082002 are elevated in PTC, correlating with lymph node metastasis and serving as potential diagnostic markers. In treatment, EV-ncRNAs contribute to overcoming therapeutic challenges. For instance, exosomal miR-1296-5p is implicated in radioiodine refractory PTC by targeting the Na+/I− symporter. EVs also serve as efficient delivery vehicles for therapeutic agents, such as siRNA and chemotherapeutic drugs, enhancing targeting and reducing side effects. Engineered exosomes carrying miRNAs like miR-152 and miR-30c-5p demonstrate potential in inhibiting tumor growth and metastasis, highlighting their role as innovative therapeutic tools in TC management. Therefore, EV-derived ncRNAs hold significant diagnostic and therapeutic potential in TC, serving as biomarkers for early detection and enhancing treatment efficacy through targeted delivery.

Stem cell-derived EVs are very efficient carrier platforms for tumor therapy due to their good biosafety, carrying properties, ease of modification, and amplification [83, 84]. MiR-152 encapsulated in bone marrow mesenchymal stem cell (BMSC) exosomes was able to significantly inhibit B-CPAP and TPC-1 cell proliferation, and migration by targeting DPP4, showing that miR-152-overexpressed BMSC exosomes could be used as an effective strategy for TC treatment [85]. Zheng et al. first showed that miR-30c-5p deletion was capable of inducing PELI1 aggregation to mediate cell proliferation and migration of PTC cells through activation of the PI3K/AKT pathway [86]. Then, they further constructed engineered human umbilical cord mesenchymal stem cells (HUCMSCs)-EVs with overexpressed miR-30c-5p that could significantly inhibit the growth of PTC cells by downregulating PELI1 expression and inhibiting the growth of PTC cells both in vitro and in vivo. Thus, the study similarly confirmed the efficacy and feasibility of miR-engineered hUCMSC-EVs for PTC therapeutic use (Fig. 4).

## Limitations and perspectives

In a general overview, EVs are important regulators of tumor behavioral remodeling. EV-ncRNAs are an important mechanistic type of epigenetic modifications that play a remarkably prominent role in the growth, proliferation, metastasis, immune regulation, diagnosis, and treatment of TC. However, many potential issues need to be addressed regarding the role of EVs in TC, including EV-related mechanisms and potential clinical applications.

First, the mechanism of EVs in TC is not yet complete and there are still many points worth noting. EVs are widely involved in various physiological and pathological processes, such as immune response, apoptosis and senescence, cell metabolism, tumor cell proliferation and invasion, and EMT [87]. Most of the relevant ncRNA epigenetic regulation is mostly focused on miRNAs. LncRNAs, circRNAs, and other less popular RNA species are not reported much at present. For example, piRNAs are small ncRNAs that play crucial roles in regulating gene expression and maintaining genomic integrity, especially in germ cells. snRNAs are essential for the splicing of pre-mRNA, ensuring proper mRNA processing. Despite their important biological functions, the roles of piRNAs and snRNAs in TC, particularly within the context of exosomes, remain largely unexplored. This limited availability of research is why these ncRNA types were not included in our analysis. And, compared to the more common tumor types of breast, liver, and gastric cancers, the number of EVs studied in TC is relatively small. That is, the types, abundance and multiple mechanisms of EV-derived lncRNAs and circRNAs in TC are not comprehensive enough. In contrast, the thyroid is an endocrine organ that secretes its hormones and is regulated by human hormones [88]. Changes in hormone levels have a major role in shaping TC TME, but there are few reports on the association among hormones, EVs, and TC [89]. In immune studies, EVs may serve an important role in antigen presentation, macrophage polarization, T cell activation, and immune remodeling. However, the immune regulation of TC by EV-ncRNAs is currently reported mainly in macrophage polarization, and little else is known. In this regard, comprehensive multi-omics sequencing and mechanistic validation are likely to address these issues and enable the identification of novel targets and mechanisms of action.

Second, all current primary studies of EV-ncRNAs in TC are in basic research and retrospective clinical studies, indicating that EV-ncRNAs are still a long way from clinical applications in TC. In diagnosis and treatment, researchers have focused more on predictive models constructed based on single EV-ncRNA or multiple EV-ncRNAs for application scenarios in TC prognosis assessment, treatment monitoring, and drug resistance treatment [90, 91]. However, there is a relative lack of sufficient sample size to validate the diagnostic value of EV-ncRNAs in certain studies. Moreover, these studies were retrospective rather than prospective, and the reliability and validity of EV as a tumor biomarker, its practical application prospects, and guidance value in the clinic need to be exhaustively determined. In terms of therapeutics, exosomes have a lipid bilayer structure that can efficiently load hydrophobic and hydrophilic drugs and are particularly suitable for the encapsulation of nucleic acid-based drugs [92]. EV-encapsulated drugs, including proteins, nucleic acid-based drugs, and chemotherapeutic drugs, display great promise for clinical applications with their unmissable advantages such as good targeting and biocompatibility. We also note that EVs are reported to be utilized as nanocarriers of tumor therapeutics for the chemotherapy of TC. However, no regard to which cell type exosomes are applied, their complex composition, uncertain biological functions, quality control, high cost, and even immunogenicity and safety issues, will more or less limit their clinical application.

Lastly, the formation of tumor drug resistance is currently considered to be a multifactorial process, including increased drug efflux, drug inactivation, altered drug targets, and inhibition of apoptosis [93]. EVs are involved in these resistance factors in many types of tumors, however, there are few reports on EVs in TC resistance therapy [94]. Currently, all studies on EV-ncRNAs in TC are still in the preclinical stage, but follow-up basic and clinical studies are still warranted to peek and support the clinical value of EVs and EV-ncRNAs. It is believed that with the expanding investigation of exosomes and the updating of basic theories and technologies, EV-ncRNAs will bring more application scenarios and values in the diagnosis and treatment of TC.

Despite the promising potential of EV-derived ncRNAs in TC diagnosis and treatment, several challenges remain in their clinical translation. One emerging perspective is the integration of artificial intelligence and machine learning to enhance the analysis and interpretation of EV-ncRNA data. By utilizing artificial intelligence algorithms, researchers can identify complex patterns and correlations in large datasets, which may not be apparent through traditional analysis methods. This approach could significantly improve the accuracy of biomarker discovery and facilitate the development of predictive models for patient stratification and personalized treatment plans. Furthermore, the heterogeneity of EVs poses a challenge in standardizing their isolation and characterization. Advances in microfluidics and nanotechnology could provide more precise and efficient methods for EV isolation, allowing for a more detailed understanding of their composition and functional roles. These technological innovations could also enable the development of point-of-care diagnostic tools, making EV-ncRNA analysis more accessible in clinical settings. Incorporating these cutting-edge technologies into EV research could accelerate the transition from bench to bedside, ultimately improving patient outcomes in TC management. As the field progresses, interdisciplinary collaboration will be essential to overcome current limitations and fully realize the potential of EV-derived ncRNAs in TC oncology.

Finally, understanding the clinical applicability of exosome-derived ncRNAs in TC is crucial for advancing their role in diagnosis and therapy. To ensure the reliability and consistency of EV-ncRNA analyses, it is essential to establish standardized protocols for the collection and storage of biological samples, such as blood, urine, and saliva. These protocols should optimize conditions to maintain the stability of EV-ncRNAs. Moreover, the development and implementation of precise and reproducible detection methods, utilizing high-throughput sequencing and advanced bioinformatics tools, can significantly enhance the accuracy of EV-ncRNA profiling, enabling the identification of highly specific biomarkers. Furthermore, designing large-scale, multicenter clinical trials is vital for validating the clinical utility of EV-ncRNAs. These trials should encompass diverse patient cohorts to ensure broad applicability and consider the specificity of different TC subtypes to assess the effectiveness of EV-ncRNAs across various clinical contexts. By addressing these aspects, we aim to expedite the clinical translation of EV-ncRNAs, ultimately offering more precise diagnostic and personalized therapeutic options for TC patients.

## Conclusion

In summary, EV-ncRNAs from different cell sources can trigger the regulatory role of ncRNAs in recipient cells of TC by information exchange. There is spatial and temporal variability in the expression abundance, pattern, and genomic characteristics of EV-ncRNAs in different TC stages. More importantly, epigenetic regulation mediated by EV-ncRNAs is involved in TC proliferation, EMT, metastasis, and immune regulation, and are promising candidates for TC diagnosis and targeted therapy. Despite the promising potential of EV-ncRNAs, challenges remain in their clinical translation, including the need for comprehensive mechanistic studies and larger-scale clinical trials. Advances in technology and interdisciplinary collaboration will be essential to fully harness the potential of EV-ncRNAs, paving the way for innovative diagnostic and therapeutic strategies that could significantly improve TC management.

## Data Availability

Data sharing is not applicable to this article as no new data were created or analyzed in this study.
